# Particulate Matter Oxidative Potential from Waste Transfer Station Activity

**DOI:** 10.1289/ehp.0901303

**Published:** 2009-12-17

**Authors:** Krystal J. Godri, Sean T. Duggan, Gary W. Fuller, Tim Baker, David Green, Frank J. Kelly, Ian S. Mudway

**Affiliations:** 1 Environmental Research Group, MRC-HPA Centre for Environmental Health, School of Biomedical and Health Sciences, King’s College London, London, United Kingdom; 2 Division of Environmental Health and Risk Management, School of Geography, Earth and Environmental Sciences, University of Birmingham, Edgbaston, Birmingham, United Kingdom

**Keywords:** antioxidants, iron, meteorology, oxidative potential, respiratory tract lining fluid, source apportionment, waste transfer sites

## Abstract

**Background:**

Adverse cardiorespiratory health is associated with exposure to ambient particulate matter (PM). The highest PM concentrations in London occur in proximity to waste transfer stations (WTS), sites that experience high numbers of dust-laden, heavy-duty diesel vehicles transporting industrial and household waste.

**Objective:**

Our goal was to quantify the contribution of WTS emissions to ambient PM mass concentrations and oxidative potential.

**Methods:**

PM with a diameter < 10 μm (PM_10_) samples were collected daily close to a WTS. PM_10_ mass concentrations measurements were source apportioned to estimate local versus background sources. PM oxidative potential was assessed using the extent of antioxidant depletion from a respiratory tract lining fluid model. Total trace metal and bioavailable iron concentrations were measured to determine their contribution to PM oxidative potential.

**Results:**

Elevated diurnal PM_10_ mass concentrations were observed on all days with WTS activity (Monday–Saturday). Variable PM oxidative potential, bioavailable iron, and total metal concentrations were observed on these days. The contribution of WTS emissions to PM at the sampling site, as predicted by microscale wind direction measurements, was correlated with ascorbate (*r* = 0.80; *p* = 0.030) and glutathione depletion (*r* = 0.76; *p* = 0.046). Increased PM oxidative potential was associated with aluminum, lead, and iron content.

**Conclusions:**

PM arising from WTS activity has elevated trace metal concentrations and, as a consequence, increased oxidative potential. PM released by WTS activity should be considered a potential health risk to the nearby residential community.

Waste transfer stations (WTSs) represent a hybrid of traffic and industrial microenvironments. These sites experience high volumes of heavy-duty diesel trucks and are often situated in densely populated urban centers (Gil and Kellerman 1993). These facilities serve as the link in the waste management system between the waste collection program and final disposal: Street waste collection vehicles discharge loads, avoiding uneconomic travel to distant landfill sites. Although this approach effectively decreases the overall air quality burden the waste management system poses on an urban center, communities in proximity to WTSs suffer from enhanced traffic flow and increased particulate matter (PM) levels arising from vehicular tailpipe/nontailpipe emissions and dust releases from laden trucks (Fuller and Baker 2001; Restrepo et al. 2004). Studies conducted in New York City (Maciejczyk et al. 2004; Restrepo et al. 2004) and London (Fuller and Baker 2008) have reported that the highest PM concentrations in these urban centers occur at sampling sites influenced by WTS-related emissions.

Concerned citizens inhabiting urban communities with WTSs across the United States have raised questions to the WTS working group of the National Environmental Justice Advisory Committee about the impact of these facilities on the surrounding communities; degraded health and environmental conditions were highlighted, as well as wider negative impacts on the community. The consequence of the environmental disamenities of these facilities has resulted in disproportionate clustering in low-income areas in the United States [National Environmental Justice Advisory Council) (NEJAC) 2000]. Similar studies in the United Kingdom have identified a disproportionately high number of industrial sites, including WTSs in socioeconomically deprived regions (Higgs and Langford 2009; Walker et al. 2005).

The relationship between extended air pollutant exposure and increased prevalence of asthma symptoms has been well documented (Romieu et al. 1995; Schwartz et al. 1993; Studnicka et al. 1997; Sunyer et al. 1997). In New York City, asthma hospitalization rates were stratified by the geographic location within each of the city’s boroughs. Children living in the Bronx, specifically the South Bronx, suffered from hospitalization rates for asthma 70% and 700% greater than the average for New York City and New York state, respectively (New York City Department of Health 1999). Sources contributing to adverse air quality in South Bronx include industrial facilities and vehicular traffic (Jackson 1995; Maantay 2007). The 19 WTSs housed in this low-income and densely inhabited community area also contributed significantly to increased PM exposures, with children experiencing elevated exposures due to the proximity of these facilities to schools, playgrounds, and residential buildings (Maciejczyk et al. 2004). To date, it is not clear whether such health effects are simply due to residents experiencing elevated PM concentrations or whether the toxicity of PM derived from WTS-related activities is elevated above that of urban PM.

Increasing interest has developed in assessing which PM physical and chemical characteristics specifically contribute to their observed toxicity. Recent literature has suggested that transition metals (Stohs and Bagchi 1995), organic species (polycyclic aromatic hydrocarbons and quinones) (Squadrito et al. 2001), and endotoxin (Thorne 2000) are associated with increased PM toxicity. The capacity of PM to induce toxicity (in particular, to elicit a respiratory and/or systemic inflammatory response) has been proposed to be a function of its oxidative potential (Li et al. 2002; Nel et al. 2001)—that is, the ability of PM to generate reactive oxygen species directly or indirectly to engender oxidative injury to the lung (Pourazar et al. 2005; Prahalad et al. 1999). We therefore evaluated PM oxidative potential to provide a simple aggregate measure of the particulate oxidative burden in the ambient airshed influenced by WTS emissions.

The objective of this study was to examine the pattern of elevated PM concentrations at a WTS located adjacent to a densely populated community in London. We used PM source apportionment techniques and microscale meteorologic information to identify pollutant episodes attributed to specific local sources, and quantified PM oxidative potential derived from WTS activity emissions.

## Materials and Methods

### Sampling location

**Figure 1:**
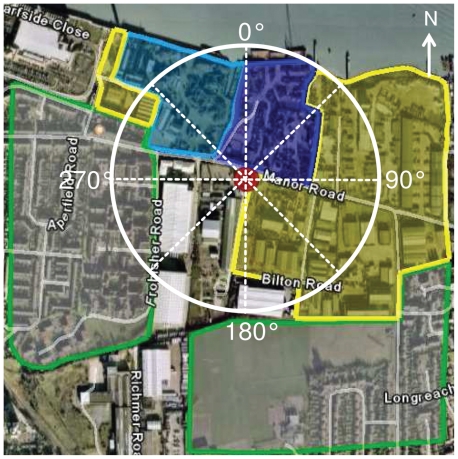
Aerial view of the WTS (red marker) located in Erith, London Borough of Bexley. The blue, yellow, and green shaded areas indicate the WTS, other industrial grounds, and residential communities, respectively. Radius of the circle corresponds to 300 m.

The Bexley 4 sampling site, located in Erith, London Borough of Bexley, was selected for investigation because it is situated at the entrance of a WTS (Figure 1). This curbside site is also situated close to a metal reclamation yard and is near high-density residential buildings. This London Air Quality Network (LAQN) site has consistently breached the European Union Limit Value/U.K. Air Quality Strategy Objective for PM_10_ (PM with an aerodynamic diameter < 10 μm), which allows 35 daily means per year > 50 μg/m^3^ [assessed as Tapered Element Oscillating Microbalance (TEOM) × 1.3]. Given the PM_10_ exceedances above regulatory limits, this sampling site was declared an air quality management area in 2001: PM_10_ concentrations were > 50 μg/m^3^ on 77 days and 116 days in 2001 and 2006, respectively (Fuller and Baker 2008).

### PM sampling

PM_10_ samples were collected daily on Teflon filters using the automated cartridge collection unit installed on the TEOM. Sampling was conducted between 5 June 2002 and 30 August 2002. To increase filter mass loading, filters were exposed repeatedly between 2 and 5 times, but only on the same day of the week as previous collections (i.e., all Monday sampling occurred on the same filter). Filters were archived at − 20°C in air-tight desiccation chambers until processed for oxidative screening and metal analysis.

### Chemicals and Chelex water preparation

All chemicals used were obtained from Sigma Chemical Company Ltd. (Poole, U.K.), Fluka (Dorset, U.K.), or Laboratory Supplies (Poole, U.K.) and were of analytical grade or better quality. Ultrapure Chelex 100 resin-treated water was employed throughout to eliminate possible background metal contamination.

### Sample preparation

Filters were extracted in high-pressure liquid chromatography–grade methanol by vortexing for 10 min before sonication using an MSE Soniprep 150 (23kHz) generator (Measuring & Scientific Equipment, London, UK) with a titanium probe at an amplitude of 5 μm for 30 sec. The methanol extract was dried under nitrogen and then resuspensed to 150 μg/mL in Chelex resin-treated water containing 5% methanol. Sample masses were determined from averaged TEOM measurements over the multiday exposure period.

### Assessment of oxidative potential

We assessed the oxidative burden associated with collected PM by measuring antioxidant depletion using a synthetic human respiratory tract lining fluid (RTLF) model as described by Mudway et al. (2004). The acellular RTLF model provides a highly repeatable and rapid method of quantifying PM oxidative potential (Ayres et al. 2008). Assessment of oxidation by this assay is limited to intrinsic and nonlatent redox components of PM: transition metal and quinone-dependent reactions.

Particle-free and known particle controls were run in parallel with filter samples to ensure interexperiment standardization. A residual oil fly ash sample, supplied by the U.S. Environmental Protection Agency (EPA), served as a positive control. A model carbon black particulate sample (M120) was selected for the negative PM control.

To demonstrate the association between PM_10_ transition metal content with oxidative potential, we performed metal chelation experiments. PM samples (50 μg/mL) in the composite 200-μM antioxidant RTLF solution were incubated for 4 hr (37°C) in the presence of diethylenetriamine-pentaacetic acid (DTPA), a strong copper [Cu(II)] and iron [Fe(III)] chelator (Buettner and Jurkiewicz 1996). Experiments were performed on pooled Monday–Friday PM suspensions and separately on Saturday and Sunday samples because of the limited sample availability. Antioxidant concentrations were determined after incubations.

### Determination of total and reduced bioavailable Fe pool

The total soluble ferrous ion pool in PM suspensions was determined using the chromogenic chelator bathophenanthroline disulfonate (BPS). BPS binds Fe(II) to form a complex that absorbs strongly at a wavelength of 535 nm, permitting the quantification of the soluble Fe(II) (Nilsson et al. 2002). The total soluble Fe pool concentration was quantified by incubating PM suspensions (100 μg/mL) at room temperature for 30 min in the presence of ascorbate (AA; 10 mM) and BPS (50 mM); AA reduces Fe(III) to Fe(II), allowing formation of Fe(II)–BPS complex, which was then measured. A standard curve (0–50 μM) of ferrous ammonium sulfate was used to determine sample Fe concentrations. Fe(II) concentrations were quantified similarly to total Fe; however, AA was not included in the incubation solution. Fe(III) was determined by subtracting the measured Fe(II) from total soluble Fe concentrations.

### Total metal screening

EMC Environmental Ltd. (Cumbria, UK) measured total metal concentrations in the PM. Samples were digested in nitric acid/peroxide and analyzed by inductively coupled plasma mass spectrometry (ICP-MS) by an EMC in-house procedure based on U.S. EPA Compendium Method 1O 3.1 and IO 3.5.

### Semicontinuous measurements

TEOM PM_10_, nitrogen oxides (NO_x_), and meteorologic [wind speed, wind direction (WD), temperature] measurements were made with 15-min resolution at the Bexley 4 sampling site. NO_x_ was measured with a Monitor Labs (Englewood, CO, USA) 9841B analyzer, and concentrations were scaled using test results from a nitric oxide calibration gas and a zero air source. Both the NO_x_ and TEOM instruments were subject to United Kingdom Accreditation Service–accredited biannual audits to ensure traceability to national metrology standards. TEOM PM_10_ and NO_x_ measurements were also undertaken to the same standards at eight additional LAQN background and suburban sites: Bexley 1, Bexley 2, Greenwich 4, Hounslow 2, North Kensington 1, Richmond 2, Thurrock 1, and Tower Hamlets 1 (LAQN 2009).

### PM_10_ source apportionment

PM_10_ concentrations were fractionated into source components over the campaign period: the total regional PM_10_ background concentration and that local to the Bexley 4 sampling site. This total PM_10_ background was further subdivided into background primary and background natural and secondary fractions using NO_x_ measurements also collected at these background/suburban sites. Background PM_10_ associated with background NO_x_ categorized the primary contribution. The remaining background PM_10_, not related to NO_x_, comprised the natural and secondary PM_10_ fraction. Further details regarding the methodology employed have been described previously (Fuller and Green 2006; Fuller et al. 2002). Total background PM_10_ estimates were then used to quantify the PM_10_ fraction measured at Bexley 4 related to local activities. Unfortunately, NO_x_ measurements at Bexley 4 were not available during the campaign to further apportion local PM_10_. As a result, local PM_10_ concentrations at the sampling site represent PM_10_-associated primary (local tailpipe emissions, vehicular-induced particulate resuspension, and tire and brake wear) and nonprimary (industrial activity) sources.

### Statistical analysis

All statistical analysis was performed using SPSS statistical program for Windows, version 15 (SPSS Inc., Chicago, IL, USA). Data are presented as the mean ± 1 SD. Data variability within groups of equal sample sizes was quantified using one-way ANOVA post hoc multiple testing with the Student Newman–Kuels test. Pearson correlations were used to assess the strength of correlations, which were considered significant for *p-*values < 0.05.

## Results

### PM source apportionment

**Figure 2:**
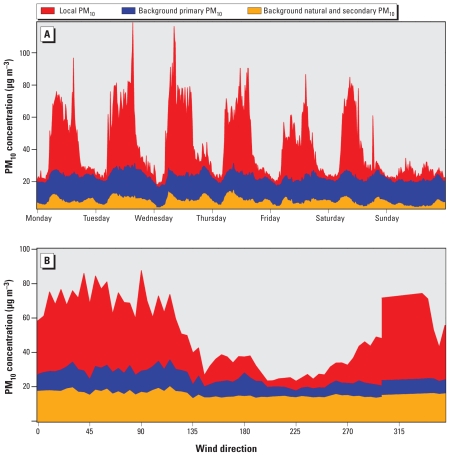
Source-apportioned PM_10_ fractions 15 min for 5 June–30 August 2002 averaged by (*A*) day of the week and (*B*) 5° WD bins at the sampling site.

The local PM_10_ fraction exhibited a clear diurnal trend from Monday to Saturday, achieving maximum concentrations during working hours. Local PM_10_ concentrations 15-min averaged on Monday-Friday, Saturday, and Sunday were 22.5, 18.2, and 4.8 μg/m^3^, respectively (Figure 2A). Locally generated PM_10_ was highly sensitive to WD: Wind originating with a bearing of 280–150°, as measured at the site, was exposed predominantly to emissions from industrial facilities (mainly the WTS) and the roadway (Figure 2B). During periods of industrial (WTS) emission exposure, the local PM_10_ fraction increased significantly (35.7 ± 9.4 μg/m^3^ average) compared with residential emission exposure (9.4 ± 5.2 μg/m^3^ average).

### PM oxidative potential

**Figure 3:**
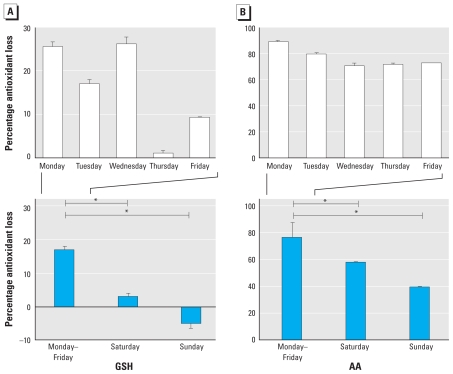
Percentage loss of GSH (*A*) and AA (*B*) relative to the 4-hr particle-free control concentration in antioxidant solutions incubated for 4 hr with PM (50 μg/mL) extracted from Teflon filters collected daily (Monday–Sunday). All filter extracts were run in triplicate. Values are mean ± SD. *p < 0.05 for difference between filter reactivity.

We observed significantly greater PM oxidative potential on weekdays per unit mass, with a decline in PM reactivity on Saturday and the lowest activity observed in the Sunday sample (Figure 3). Although PM oxidative potential was highest on weekdays, considerable variation existed within Monday–Friday samples. AA losses ranged from 71 to 89%, and glutathione (GSH) losses ranged from 1 to 26%.

### Metal analysis

Filters were assessed for the following metal concentrations, which were then standardized by the PM mass collected on each filter: aluminum (Al), arsenic (As), cadmium (Cd), Cu, Fe, lead (Pb), magnesium (Mg), platinum (Pt), tungsten (W), and zinc (Zn) (Table 1). Of these, Pt, W, and As were below the limit of detection. Cd and Cu were present at very low levels; all quantities were near the instrument detection limits (< 0.05 μg per microgram PM) after subtraction of background filter concentration and thus were considered unsuitable for inclusion in the correlation analysis.

Fe and Pb concentrations followed the pattern of industrial activity: higher concentrations on Monday–Friday, intermediate concentrations on Saturday, and further decreases on Sunday. Similar to antioxidant concentrations, significant interweekday variability was observed for each of these metal species.

### Bioavailable Fe

**Figure 4:**
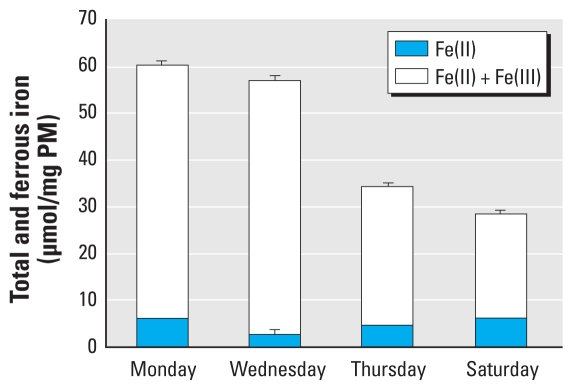
Mean total and ferrous bioavailable Fe concentrations in PM_10_ with the associated SD of sample replicates (*n* = 3).

Metal species associated with PM exist in various forms, each with differing abilities to drive reactions. In the lung, as it is likely that only bioavailable (soluble and surface-associated) metals govern PM oxidative potential, it was of interest to quantify the bioavailable fraction of Fe. Because of the limited availability of sample material, this analysis was possible only on four (Monday, Wednesday, Thursday, and Saturday) of the seven filter samples (Figure 4). AA loss from the RTLF was partially explained by its oxidation during the reduction of Fe(III) to Fe(II) (Davies 1992; Miller and Aust 1989). Thus, samples with high Fe(II) metal content were expected to be the most reactive; measured weekday samples were more active compared with the Saturday samples across the sampling period.

### Metal chelation analysis

Transition metal–induced PM oxidative potential was confirmed by metal chelation experiments. In all cases, PM_10_ incubation with an excess of DTPA completely blocked PM-dependent AA and GSH oxidation (Table 2). The relationship between the extent of antioxidant depletion and the concentration of metals on the PM filters was examined using the Pearson correlation. A significant association was apparent between the extent of AA and GSH from the composite antioxidant solution after incubation with model and ambient PM (*r* = 0.78, *p* = 0.039, *n* = 7). Moreover, ICP-MS analysis revealed significant associations between the degree of AA loss and total Pb (*r* = 0.88, *p* = 0.009) and Fe (*r* = 0.80, *p =* 0.032) metal concentrations. In contrast, the loss of GSH was not related to Fe but rather to Al (*r =* 0.76, *p =* 0.048) and Pb (*r =* 0.89, *p =* 0.008) concentrations. Notably, the association between the antioxidant loss and Pb reflected an underlying association between this trace metal with Fe (*r =* 0.87, *p =* 0.011) and Al (*r =* 0.84, *p =* 0.019).

### WD and correlation analysis

**Figure 5:**
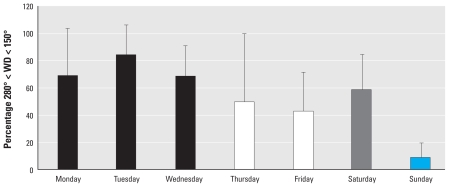
Percentage (mean ± SD) of daily averaged 15-min resolution WD measurements with a bearing of 280–150°, corresponding to the frequency at which the sampled air originated predominantly from over the industrial facilities. Bar color indicates the four emission scenarios during the sampling campaign: black, weekday samples when emissions were primarily emitted from the WTS; gray, weekend sample influenced predominantly by the local WTS; blue, weekend sample exposed primarily to residential emissions; white, remaining samples representing mixed residential and WTS emission cases.

Weekday WTS emissions all exhibited an equivalent diurnal profile and maintained an approximately constant magnitude. Thus, the resulting PM oxidative potential was not expected to fluctuate from Monday to Friday. However, the observed variability in PM oxidative potential (comprising antioxidant depletion, the bioavailable Fe pool, and total metal concentrations measurements) suggested that regardless of proximity, the sampling site was not continually exposed to WTS emissions. This was likely due to oscillations in WD. As a result, exposure scenarios were defined for each day of the week during the sampling period to account for the percentage of time that the sampled PM_10_ was influenced by fugitive WTS emissions. Fifteen-minute averaged WD measurements were considered for each individual day included in the bulk filter exposures. WD measurements between 280° and 150° indicated that sampled PM_10_ was influenced by industrial sources, whereas remaining bearings were classified as residential-derived PM_10_ (Figure 5). This WD grouping method results in three cases, each presenting different emission scenarios (Table 3): *a*) Monday–Wednesday, weekday filters when the sampled emissions were primarily emitted from the WTS; *b*) Saturday, a weekend sample influenced predominantly by the local WTS; and *c*) Sunday, a weekend sample that represented a filter primarily exposed to residential emissions. The remaining sampling days served as mixed residential and WTS emission sampling cases.

**Figure 6:**
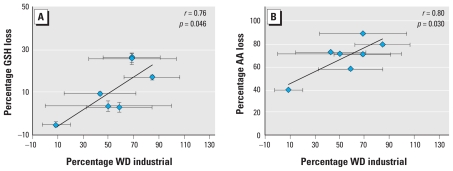
Correlation between the percentage of GSH (*A*) and AA (*B*) relative to the 4-hr particle-free control concentration and the frequency sampled air was transported over industrial facilities (percent WD industrial given by a WD with a bearing of 280°–150°) (*n* = 7). Values shown are mean ± SD.

The days of the week predominantly influenced by WTS emissions corresponded to a greater percentage loss of AA (*r =* 0.80, *p =* 0.030) and GSH (*r =* 0.76, *p =* 0.046) concentrations relative to 4-hr particle-free controls (Figure 6). Days of the week (Monday–Wednesday) with bulk filter exposures that experienced the greatest influence of WTS emissions corresponded with the highest levels of antioxidant loss. Locally generated PM_10_ concentrations were positively associated with the frequency with which WTS emissions were sampled during each bulk filter exposure.

When considering only days when the WD carried emissions predominantly originating from the WTS area to the sampling site, the percentage of antioxidant depletion was higher on weekdays (Monday–Wednesday) than on Saturday. Weekday local PM_10_ fraction concentrations during working hours remained approximately constant. Moreover, weekday levels were not statistically different from concentrations measured on Saturdays. However, PM metal composition did vary significantly between weekday and Saturday samples. The lower Saturday metal concentrations were likely responsible for the decreased PM oxidative potential. Comparison of Saturday versus Sunday PM (weekend periods sampling industrial and residential emissions, respectively) revealed that antioxidant losses were higher on Saturday, which was potentially attributed to a change in WD and a decrease in locally produced PM_10_.

## Discussion

Elevated PM concentrations have been reported in proximity to WTS (Fuller and Baker 2008; Maciejczyk et al. 2004; Restrepo et al. 2004), but no study has yet examined whether there is a basis for believing PM from these industrial emissions may have enhanced toxicity, based on their chemical composition. Questions have also been voiced by concerned residents in these areas regarding the environmental disamenities resulting from the poor air quality including degraded health and limited economic growth (NEJAC 2000). To investigate the raised health concerns and to examine whether there was any basis for believing PM from such facilities might display enhanced toxicity, we examined both the composition and oxidative properties of PM generated by WTS activity. Incorporation of PM oxidative potential into a receptor model source apportionment analysis is a novel aspect of this study.

A weekly pattern of local PM_10_ concentrations was observed at the sampling site: Elevated concentrations were found on weekdays and Saturdays, with much lower concentrations on Sundays. The PM_10_ mass concentration difference between weekday and Sunday samples was expected, because the WTS was closed on Sundays. This general trend also extended to oxidative potential measurements. Intercomparison of weekday oxidative potential levels revealed variation regardless of the continual WTS operation during these days. The extent that industrial emissions influenced sampled PM_10_ concentration, as predicted by WD, was used to explain this weekday variation. Moreover, elevated PM oxidative potential was established when WTS emissions predominately contributed to PM sampled at this site. Additionally, the measured PM oxidative potential was likely attributable to the compositional profile of the local PM_10_ fractions, given its high sensitivity to WD.

The oxidative potential of PM derived from WTS was compared with the PM total metal and bioavailable Fe content. Correlations between total transition metals and antioxidant depletion produced significant associations between metal species and GSH (Al, Pb) and AA (Fe, Pb) depletion. Moreover, the concentration of Pb was strongly associated with both Fe and Al, suggesting a common source(s). Total Fe and Pb exhibited a significant difference between weekday, Saturday, and Sunday concentrations following the pattern of locally produced PM_10_ at the sampling site.

Cu was not identified as a predicator of PM oxidative potential, unlike results from previous studies (Künzli et al. 2006; Mudway et al. 2004). Differences in the traffic profile are likely accountable for this discrepancy: Cu is a marker of light-duty vehicle brake wear, whereas barium and antimony are characteristic of brake wear from heavy-duty vehicles (Sternbeck et al. 2002). The vehicle fleet servicing the WTS comprised predominantly the latter type, unlike the traffic profiles of the previously mentioned studies.

Bioavailable Fe (BPS-mobilized Fe, as opposed to total Fe), is the key determinant of the PM-associated Fe pool that would be available *in vivo* to drive damaging oxidations (Aust et al. 2002; Duggan 2007). These BPS measurements of soluble Fe also provide information regarding oxidation state. This is useful, as PM oxidative potential is partially explained by the reduction of Fe(III) to Fe(II), and therefore samples with high Fe(III) metal detail are expected to be most reactive. Low-molecular-weight antioxidants found in the RTLF model and lung are able to rapidly reduce Fe(III) complexes. Upon ferric ion reduction, AA is simultaneously oxidized. In addition, the superoxide formed in these reactions will further drive AA and GSH oxidation. Moreover, selection of BPS as a chelator has biological relevance: The stability constant of BPS for Fe(III) is 1 × 1022 M (Nilsson et al. 2002), which is lower than that for transferrin (1 × 1028 M) and albumin (Halliwell and Gutteridge 1999; Halliwell et al. 1999), providing a measure of the Fe pool likely to be associated with low molecular weight ligands *in vivo* and hence its catalytic activity.

Bioavailable Fe content was considered for a subset of exposed samples. Weekday (Monday and Wednesday) samples were found to have the highest ferric ion content. This trend of elevated weekday Fe(III) concentrations also corresponded with the highest PM oxidative potential measurements reflective of WTS contributions to the sampling site rather than residential emissions. As the contribution of WTS emissions to sampling site declined, so too did the PM_10_ bioavailable iron pool. Because only four samples were available for this type of analysis, it was not appropriate to attempt soluble ferric ion content correlation analysis with WTS emission exposure (280° < WD < 150°) and antioxidant depletion.

Organic PM content, including polycyclic aromatic hydrocarbons, quinones, and endotoxin, were not measured in the present study, although they can also trigger the generation of reactive oxygen species, once inhaled, through metabolism and the induction of inflammation (Ayres et al. 2008). Of these components, only particulate quinone content would have influenced the measured PM oxidative potential in the acellular RTLF model because of their capacity to redox cycle (Squadrito et al. 2001). Thus, the measured PM oxidative potential in the RTLF model is restricted to the intrinsic redox active components, catalytic metals, and quinones and should not be viewed as reflecting the total activity of the sample through its interaction with the complete biological matrix. However, the metal chelator experiments demonstrated that quinone-driven oxidative activity of WTS-derived PM was low (Table 2). No significant antioxidant depletion was measured when PM_10_ samples were incubated with DTPA. This demonstrates that PM catalytic metal content explained much of the intrinsic capacity of these samples to catalyze damaging oxidation reactions.

## Conclusion

PM_10_ exceedances at the sampling site were the result of WTS emissions linked to site activity. Detailed PM chemical characterization indicated that these high PM_10_ mass concentrations contained elevated transition metal levels. Concentrations of total Fe, Al, and Pb were especially high; WTS soluble Fe emissions existed predominantly in the form of Fe(III). Assessment of the WTS PM oxidative potential revealed that it was associated with increased transition metal concentrations. Thus, PM_10_ generated by WTSs may be a potential health risk to neighboring communities. The resident population is exposed to a high PM mass concentration on a recurrent basis, and particulate emitted by these industrial emissions may have a greater capacity to drive damaging oxidation reactions in the lung compared with other PM sources in urban environments.

## Figures and Tables

**Table 1:** Individual filter metal concentrations. All metal concentrations were standardized by the total PM mass collected on the filter (mg PM_10_).

Filter	Fe	Pb	Al	Mg	Zn
Monday	25.9	2.32	5.22	2.35	0.94
Tuesday	10.5	1.35	2.10	1.15	0.65
Wednesday	13.9	1.59	3.61	1.91	0.94
Thursday	16.2	1.02	2.29	0.77	0.00
Friday	5.04	1.20	1.73	1.11	0.38
Saturday	9.14	1.14	1.91	0.83	0.30
Sunday	0.00	0.29	1.91	1.65	0.64

**Table 2:** Details of DTPA metal chelation incubations with PM_10_ samples.

	Percent AA loss		Percent GSH loss	
Filter	(−DTPA)	(+DTPA)	(−DTPA)	(+DTPA)
Monday–Friday	61.3 ± 1.25	1.56 ± 1.56	19.6 ± 6.20	ND
Saturday	50.9 ± 1.26	0.38 ± 1.40	1.10 ± 5.43	ND
Sunday	44.3 ± 2.33	3.08 ± 3.61	12.7 ± 1.64	ND

ND, not detected. (−DTPA) and (+DTPA) represent incubations without and with DTPA, respectively. The percentage of antioxidant losses are expressed relative to the zero time point particle-free control, as incubations with DTPA eliminated the background auto-oxidation seen in the particle-free controls.

**Table 3:** Summary of PM_10_ toxicity analysis grouped by WD.^a^

Case	Filter	Source	% AA loss	% GSH loss	PM_10_ (mg)	Fe	Al	Pb
1	Mon–Wed	Industrial	79.8 ± 1.2	23.0 ± 2.2	2.71 ± 0.75	16.80 ± 6.61	3.65 ± 1.27	1.50 ± 0.51
2	Sat	Industrial	57.6 ± 1.2	3.0 ± 2.4	3.14	9.14	1.91	1.14
3	Sun	Residential	39.5 ± 0.6	−5.0 ± 1.1	1.06	0.00	1.91	0.29

a Mean weekday concentrations are presented ± 1 SD. Metal concentrations are standardized to the PM mass on the filter [μg (mg/PM_10_)].
